# Resting-State EEG Oscillatory Dynamics in Fragile X Syndrome: Abnormal Functional Connectivity and Brain Network Organization

**DOI:** 10.1371/journal.pone.0088451

**Published:** 2014-02-11

**Authors:** Melle J. W. van der Molen, Cornelis J. Stam, Maurits W. van der Molen

**Affiliations:** 1 Institute of Psychology, Developmental Psychology Unit, Leiden University, Leiden, the Netherlands; 2 Leiden Institute for Brain and Cognition. Leiden, the Netherlands; 3 Department of Clinical Neurophysiology, VU University Medical Center, Amsterdam, the Netherlands; 4 Neuroscience Campus Amsterdam, VU University Medical Center, Amsterdam, The Netherlands; 5 Department of Developmental Psychology, University of Amsterdam, Amsterdam, the Netherlands; 6 Cognitive Science Center Amsterdam, Amsterdam, The Netherlands; University of Jaén, Spain

## Abstract

Disruptions in functional connectivity and dysfunctional brain networks are considered to be a neurological hallmark of neurodevelopmental disorders. Despite the vast literature on functional brain connectivity in typical brain development, surprisingly few attempts have been made to characterize brain network integrity in neurodevelopmental disorders. Here we used resting-state EEG to characterize functional brain connectivity and brain network organization in eight males with fragile X syndrome (FXS) and 12 healthy male controls. Functional connectivity was calculated based on the phase lag index (PLI), a non-linear synchronization index that is less sensitive to the effects of volume conduction. Brain network organization was assessed with graph theoretical analysis. A decrease in global functional connectivity was observed in FXS males for upper alpha and beta frequency bands. For theta oscillations, we found increased connectivity in long-range (fronto-posterior) and short-range (frontal-frontal and posterior-posterior) clusters. Graph theoretical analysis yielded evidence of increased path length in the theta band, suggesting that information transfer between brain regions is particularly impaired for theta oscillations in FXS. These findings are discussed in terms of aberrant maturation of neuronal oscillatory dynamics, resulting in an imbalance in excitatory and inhibitory neuronal circuit activity.

## Introduction

Fragile X syndrome (FXS) is the most common inherited neurodevelopmental disorder caused by a single gene defect, and provides a unique opportunity to study the neurobiological mechanisms of brain development and cognitive function. Despite the vast literature on functional brain connectivity in typical brain development, surprisingly few attempts have been made to characterize brain network integrity in neurodevelopmental disorders. In this study we employed a systems-neuroscience approach to characterize functional brain connectivity and brain network organization in FXS males based on resting-state EEG time-series.

The neurobiological hallmark of FXS is the silencing of a single gene (FMR1) located on the X-chromosome [1], [2], resulting in reduced or absent levels of its gene product – the fragile X mental retardation protein (FMRP) [3]. Both humans and rodents with the FXS full mutation consistently display an excess of long and thin dendritic spines, resembling immature cortical networks [4]–[7]. This observation is suggestive of abnormal dendritic pruning processes, which compromise normal brain development via aberrant synaptic plasticity [8], [9]. Neurobiological studies have revealed that absent or reduced FMRP expression can be linked to imbalanced cortical excitatory (glutamatergic) and inhibitory (GABAergic) circuit activity in *fmr1* knockout mice [10], [11]. Specifically, excess signaling of glutamate receptors contributes to spontaneously occurring neuronal firing states (UP states), as well as exaggerated long-term depression [12], [13]. In typical development, long-term depression decreases synaptic strength and long-term potentiation increases synaptic strength. Both processes work in concert in response to neural signal transmission mechanisms for regulating synaptic plasticity – a key biological mechanism during brain development [14]. Disturbed glutamatergic and GABAergic activity is argued to disrupt these neurobiological processes, resulting in cortical hyperexcitability FXS [10], [15].

To date, it remains unclear how these neurobiological alterations change the functional connectivity between local and distant brain regions as well as the overall organization of large-scale brain networks. Such information is paramount to better understand how the aforementioned neurobiological changes affect neurocognitive processes and the attentional and behavioral abnormalities frequently reported in FXS [16]–[18]. Given the apparent changes in neuronal excitation and inhibition [10], [19], and the notion that glutamatergic and GABAergic circuit activity serves a critical role in the gating of neuronal oscillations and synchrony [20], investigating neuronal oscillatory activity and functional connectivity could shed light on the integrity of local and global neuronal communication in the FXS brain.

In the current study, we examined the integrity of functional brain connectivity in various spectral bands of the electroencephalogram (EEG). In addition, we employed graph theoretical network analyses, which allows for a systematic investigation of the network architecture governing neuronal oscillations. Using graph theory, the neural architecture of the brain can be parceled into networks of nodes and links. Nodes are generally referred to as processing units, whereas links represent the (anatomical or functional) connection between the nodes. The organization of nodes and links in a graph is purported to reflect the integrity and efficiency of brain networks [21], [22]. The clustering coefficient (a measure of local connectedness of a graph) and path length (for unweighted networks: the number of edges in the shortest path between two vertices in a graph) are two indices that reflect the complexity of the graph or brain network [21], and can be used to classify brain network topology. Human brain networks have been shown to resemble the ‘small-world’ properties - characterized by high clustering and short path length that optimize information transfer within the brain network [21], [22]. It has been shown that the brain becomes less random and shows increased small-world characteristics with ongoing development [23], [24]. Furthermore, it has been shown that shorter normalized path length is associated with higher levels of full-scale IQ [25], [26], suggesting that path length is crucial for information processing efficiency within the network.

Based on evidence of immature neuronal networks in both the *fmr1* knock-out mice and FXS human brain [5], and aberrant theta and alpha oscillatory power during resting-state EEG [27], we tested the following hypotheses in the current study: (1) functional connectivity was expected to be increased for theta oscillations, whereas a decrease in alpha oscillatory functional connectivity was hypothesized; (2) we anticipated to find evidence of altered organization of functional brain networks in FXS individuals, which would be more reflective of immature random organization rather than the more optimal small-world, organized topologies. In line with the more random network topologies found in early childhood [24], the anticipated pattern of findings would be a manifestation of the alleged immature state of cortical networks in FXS.

## Method

### Participants

Eight male participants diagnosed with the FXS full mutation (mean age = 26.25, SD = 8.00) and 12 healthy age-matched male controls (mean age = 26.75, SD = 4.05) participated in this study. Prior DNA testing was performed by clinical geneticists and confirmed the diagnosis of the FXS full mutation (i.e., CGG repeat size >200) in the FXS participants. The number of CGG repeats was not available for all FXS participants, but the full mutation was confirmed by prior diagnostic testing. None of the FXS participants had a prior history of epileptic seizures, or any other neurological complications. For both groups, non-verbal intelligence was assessed using the Raven Standard Progressive Matrices [28]. Raw scores were significantly lower in FXS males (mean = 21.00, SD = 8.60) than in control participants (mean = 56.25, SD = 2.38).

Average Raven-IQ of the control participants was 121.50 (SD = 25.79). IQ of the FXS participants was equivalent to an average mental age of 7.73 years (SD = 1.59). For the FXS individuals, non-verbal and verbal mental age has been previously assessed [17] using more suitable intelligence tests for individuals with developmental disorders. Performance on the Snijders and Oomen Non-Verbal Intelligence Test [29] corresponded to an average non-verbal mental age of 5.29 years (SD = 1.22), whereas performance on the Dutch version of the Peabody Picture Vocabulary Test third Edition [30] corresponded to an average verbal mental age of 9.65 years (SD = 3.20). FXS participants were recruited with the help of the Dutch Fragile X Parent Network. Control participants were recruited from or within the proximity of the university, and received course-credit or a monetary compensation for their participation. None of the participants were on medication during the experiment. All participants were reported to have intact hearing and had normal or corrected-to-normal vision.

### Ethics statement

The protocol for this study was reviewed and approved by the ethical review committee of the department of Psychology of the University of Amsterdam. Signed informed consent was obtained prior to the experiment from control participants. For the FXS participants, written informed consent was obtained from their parents or primary caregivers.

### EEG recordings and preprocessing

Closed-eye resting-state EEG was collected prior to two attentional paradigms [31], [32], and the procedure for obtaining resting-state epochs is similar to our previous EEG power study [27]. EEG recordings were collected at home locations for all FXS participants. For half of the control participants, EEG recordings were collected at home locations, for the other half at the University lab. Participants were first instructed to refrain from moving and asked to close their eyes for a 5-minute period. EEG was recorded using an EasyCap electrode cap with 26 Ag/AgCl sintered ring electrodes using the 10/20-system placement. Recording electrode positions included: Fp1, Fp2, F7, F3, Fz, F4, F8, FC1, FCz, FC2, FC6, T7, C3, Cz, C4, T8, CP1, CP2, P7, P3, Pz, P4, P8, O1, Oz, and O2. Electrodes placed at the left and right mastoids were used for linked reference. FT9 served as ground. Horizontal eye movements (HEOG) were recorded using bipolar electrodes placed at the outer canthi of the eyes. Electrodes for recording vertical eye movements (VEOG) were placed just above and under the left eye. Electrode impedances were kept below 10 kΩ. Signals were recorded with a BrainAmp DC amplifier (Brain Products) using Brain Vision Recorder software, at a sampling rate of 500 Hz and an online filter between 0.3 and 70 Hz.

**Figure 1:**
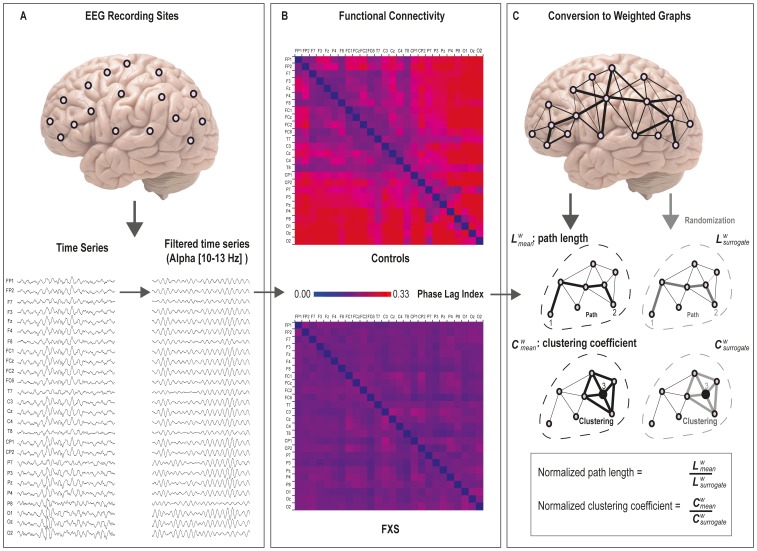
An overview of the method used to calculate weighted graphs. (A) EEG time-series from the 26 scalp electrodes were separately filtered for the delta, theta, alpha low, alpha high, beta, and gamma frequency bands. Higher alpha (10–13 Hz) is shown here as largest group differences were found for this frequency band. (B) Functional connectivity between all 26×26 electrode pairs was calculated based on the phase lag index, yielding connectivity values between 0 and 1 (higher values reflect more synchronization between electrodes). (C) When using graph theoretical analysis on EEG time series, electrodes represent “nodes” and the distance between these nodes represent the “edges” in the graph. PLI scores were used to calculate the path length (distance between the nodes) and the clustering coefficients (the degree in which nodes cluster together). In addition, a randomization procedure is employed to obtain measures independent of network size. From each original graph, random networks were derived by randomly shuffling the edge weights. Mean values of weighted graphs are then determined by dividing the original graph measures by these ‘surrogate’ measures.

Closed-eyes continuous resting-state EEG recordings were offline filtered at 0.5–50 Hz using Brain Vision Analyzer software (Version 1.05, © Brain Products) and visually inspected for artifacts (e.g., eye blinks, eyes and body movement, muscle contractions, low-quality channels). Resting state EEG was thereafter converted to six artifact free epochs containing 4096 time samples and exported to ASCII files. Subsequent analyses were performed separately for the delta (0.5–4 Hz), theta (4–8 Hz), lower alpha (8–10 Hz), upper alpha (10–13 Hz), beta (13–30 Hz), and gamma (30–45 Hz) bands, with BrainWave software v0.9.76 (developed by C.S.; freely available at http://home.kpn.nl/stam7883/brainwave.html). Figure 1 depicts an overview of the steps employed in the normalized graph analysis of the EEG time series.

### Functional connectivity: the phase lag index

Functional connectivity between all 26-electrode pairs was calculated using the phase lag index (PLI), a measure of the asymmetry of the distribution of phase differences between EEG signals. A detailed method for calculating the PLI is described in previous work [33]. In order to compute phase synchronization, the instantaneous phase of two signals needs to be determined. This can be accomplished by using the analytical signal based on the Hilbert transformation [33]. Subsequently, the PLI is obtained from time series of phase differences 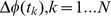 by means of:
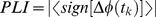
(1)in which 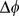 represents the phase difference and 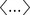 denotes the average over time *t*. The PLI performs at least as well as the synchronization likelihood [34] or phase coherence [33] in detecting true changes in synchronization but is less affected by the influence of common sources and/or active reference electrodes [35]. This is due to the fact that the zero-lag synchronization is removed from the analyses, and that the PLI only quantifies the relative phase distribution's *asymmetry*, which refers to the likelihood that the phase difference 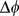 is in the interval 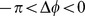 is different from the likelihood that it is in the interval 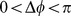. PLI values range between 0 and 1. A PLI value of zero indicates either no coupling or coupling with a phase difference centered around 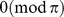. A PLI value of 1 indicates perfect phase locking at a value of 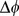 different from 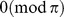. The more consistent this nonzero phase locking is, the larger is the PLI. It should be noted that the PLI does not provide an index of causal interaction between synchronized electrodes. To test the robustness of our PLI findings, we also calculated the functional connectivity based on the directed phase lag index (dPLI) [36]. In contrast to the PLI, the dPLI takes into account the asymmetric phase relations. dPLI values can range from 0 to 1. Values 0.5 reflect phase lagging, whereas values of >0.5 reflect phase leading. Similar results were obtained using the dPLI. Only the PLI results will be reported in this study, since the PLI was used as weight definition in the graph analyses.

### Graph Analysis

By using EEG time series for graph analyses, the electrodes represent nodes or vertices in the graph and the strength of the synchronization between EEG time series recorded at these electrodes can be taken as a measure of association between the vertices. This association between vertices can be assigned a weight 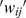 that reflects the either *strength* of the relation between vertices 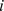 and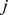 (when computing the weighted clustering coefficient) or the inverse of the strength (when computing the weighted path length). The 26 EEG electrodes represent the vertices in the graph and the matrix of PLI values between all pairs of electrodes is used to specify the association between vertex 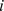 and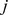. This produces weighted graphs for which a detailed description has been reported in previous work [37], [38]. Two fundamental aspects in a graph are the clustering coefficient and the path length [39]. For a vertex 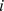 the clustering coefficient 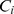 is a measure of local connectedness of a graph. The clustering coefficient represents the likelihood that neighbors of vertex 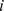 are also connected to each other. For calculating the weighted clustering coefficient, the method as described by Stam et al. (2009) was used, which states that the weights between node 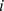 and other nodes 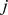 should be symmetrical 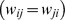 and that 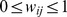
holds [37]. Both conditions are met when using the PLI as weight definition for the clustering coefficient (i.e., 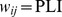). The weighted clustering coefficient of node 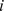 is defined as:
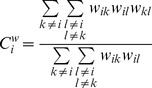
(2)Note that in equation (2) terms with 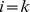, 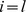, and 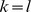 are not included. For isolated vertices (i.e., vertices that do not have any connections), the clustering coefficient is defined as 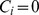 [37]. The mean weighted clustering coefficient of the total network is defined as:
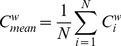
(3)In equation (3), 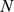 represents the number of vertices. For a given node 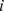 in the graph, the shortest path algorithm finds the path with the lowest cost (i.e., the shortest path length) between that node and every other node. The weighted path length represents the distance between pairs of vertices of the weighted networks and is calculated following the approach of Latora and Marchiori (2001) who define the length of an edge as the inverse of the weight [40]. The average shortest path length for node 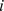 to all other nodes is defined as:
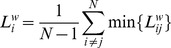
(4)In equation (4), 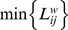 is the weighted shortest path length 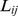 between node 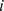 and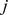, and 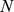 represents the number of vertices. For the weighted path length based on PLI weights, 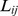, the path between nodes 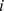 and 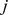 is found by minimizing the sum of the “distance weights” 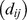 assigned to the edges on their path. Such distance transformation 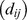 was defined as the inverse of the PLI values (i.e. 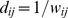). Dijkstra's algorithm (Dijkstra, 1959) was used to find the shortest path length with the lowest possible sum of the distance weights [41], [42]. Notice that 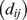 is a positive value since 1/PLI is used as the edge weight. Average path length (average of all 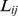) was based on the arithmetic mean. The mean weighted shortest path length can be computed by:
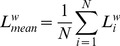
(5)In equation (5), 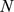 represents the number of vertices. Note that a path consists of two (or more) edges which each their own weight. A path is shorter (i.e., more efficient) when the sum of these weights 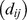 is higher.

### Normalization

As indicated in prior work [37], individual networks vary in terms of structure, edge weights, and size, which affect the graph parameters of interest (i.e., path length, clustering size, and weight dispersion). In order to obtain graph parameters that are independent of individual differences in PLI and network size, the weighted clustering coefficients 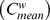 and path length 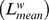 were compared to its surrogate values derived from random networks (i.e., 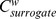 and 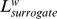). In contrast with small-world networks (which best characterize the topology of neural networks by high clustering and short average path length), random networks display low heterogeneity. That is, pairs of vertices in a random network have an equal probability of being connected [21]. These random networks are not representative for models of complex networks, such as the brain, but can be used as a null-model for comparison [21], [43]. Random networks were derived by randomly shuffling of the original edge weights. This procedure was performed for 50 randomized networks. Normalized clustering coefficients 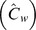 and path length 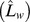 were then defined by dividing the actual values by the randomized values (i.e., 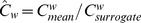 and 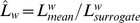). The small-world characteristics of the network can be represented by the small-world index 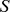 [44], which can be calculated by the following: 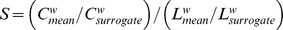. If both 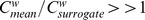 and 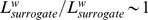, a network can be defined as a small-world network. Thus, 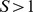 defines the “small-worldness” of a network.

### Statistical analyses

All statistical analyses were carried out using SPSS version 19. The PLI and graph measures (clustering coefficient and path length) were log-transformed (due to a non-normally distribution) and two-tailed *t*-tests for independent samples were used to assess group differences. Alpha was set at 0.05 for significance testing. Due to the small sample size of the FXS group, we ran power analyses to determine a sufficient sample size. Results showed that a minimum of 26 participants per group is required to detect a significant difference (alpha .05) with an appropriate level of power (i.e., 0.80).

## Results

### Functional connectivity as indexed by the phase lag index

**Figure 2:**
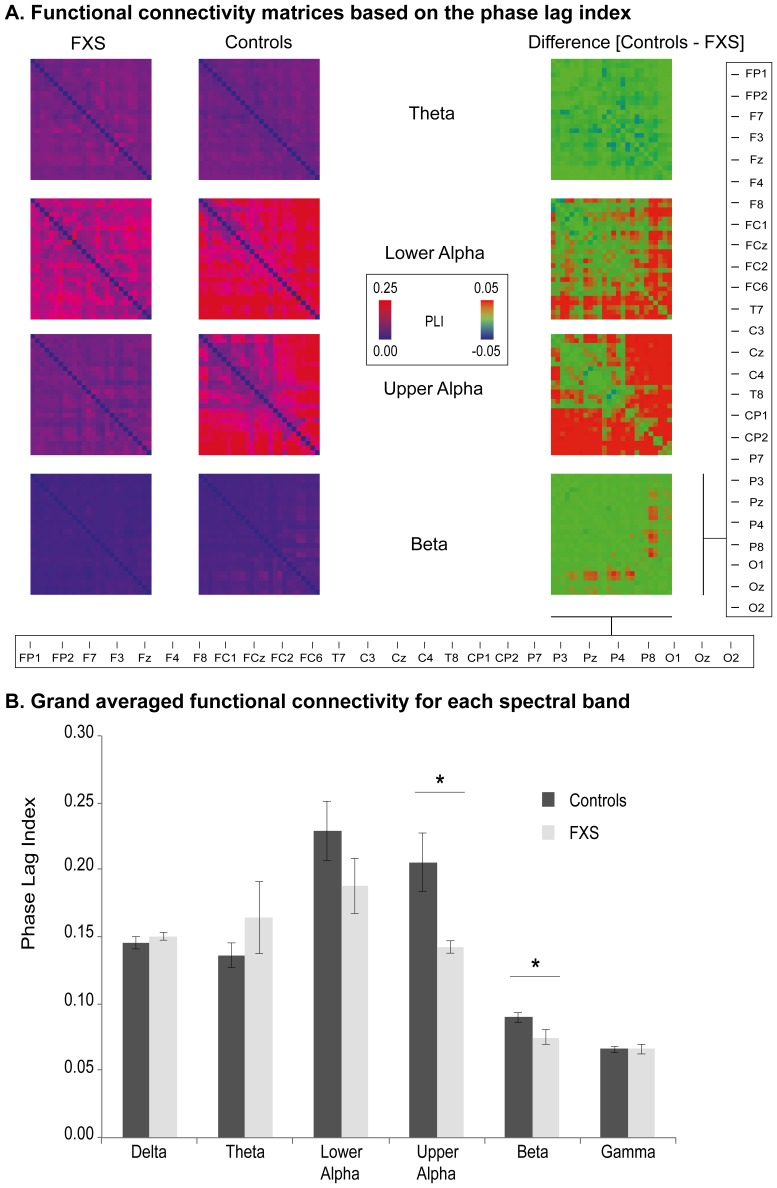
Global functional connectivity as indexed by the Phase Lag Index (PLI). (A) Functional connectivity matrices are presented for theta, alpha and beta bands, as group differences were largest for these spectral bands. (B) Group differences in PLI for the delta, theta, alpha, beta and gamma bands. As can be seen from these data, FXS males show significantly less functional connectivity in the upper alpha and beta frequency bands. Asterisks represent significant differences at *p*<.05. Error bars represent standard error of the mean.

Figure 2 depicts an overview of the functional connectivity results for the various EEG spectral bands. A first step was to analyze group differences in overall PLI values (averaged over all pairs of EEG channels) per spectral band. In panel A of Figure 2, a clear difference can be observed in the PLI values between FXS and controls for the alpha spectral band. That is, functional connectivity was lower in FXS individuals for alpha oscillations, but these differences were only significant for the upper alpha spectral band (10–13 Hz), *t*(18) = −2.56, *p* = .020, *η*_p_^2^ = .27. Since low spectral power could be an indication of confounded PLI estimation, we examined the correlations of the PLI per spectral band with their corresponding spectral power values. We only observed a negative correlation between PLI and spectral power of Beta oscillations (13–30 Hz), *r* = −.725, *p* = .04, which could possibly have underestimated the functional connectivity in the Beta band in FXS individuals. In addition, functional connectivity was lower in FXS individuals than in controls for beta oscillations (13–30 Hz), *t*(18) = −2.19, *p* = .042, *η*_p_^2^ = .21. Higher functional connectivity was observed for FXS males in the theta band (4–8 Hz), but this difference was not significant. The overall functional connectivity results suggest differences in slow (theta) and faster (alpha and beta) oscillatory networks, which could be reflective of aberrant maturational processes, since functional connectivity in the alpha and beta bands are known to reach peak values at later stages during development [45].

**Figure 3:**
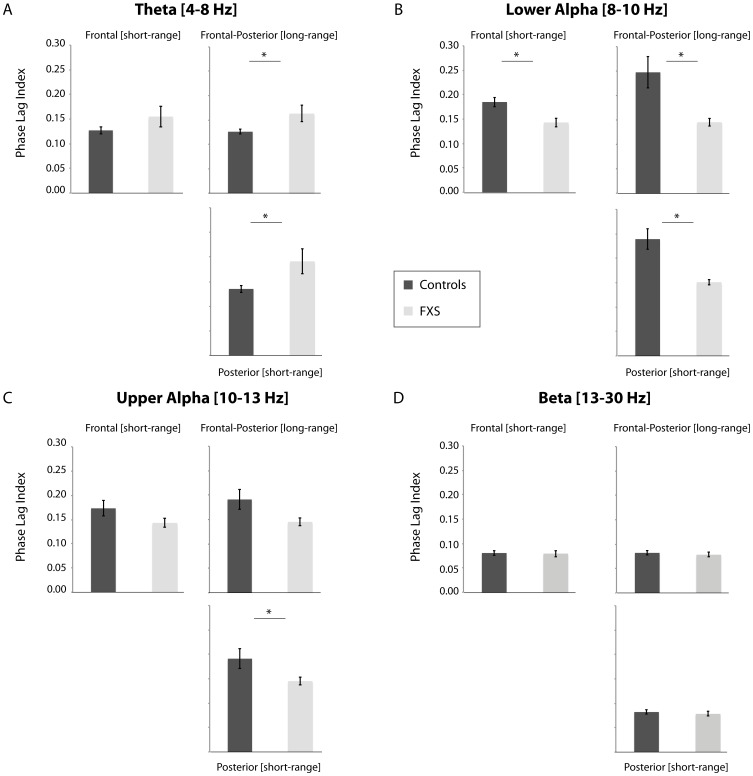
Matrices of local and long-range functional connectivity in frontal and parietal/occipital clusters for the theta and upper alpha power bands. In FXS males, significant increased local functional connectivity was found in the parietal-occipital cluster for theta oscillations, whereas a decrease in local functional connectivity was found in this cluster for alpha oscillations. A significant increase in long-range (frontal-parietal/occipital) theta functional connectivity was found in FXS males. Asterisks represent significant differences at *p*<.05. Error bars represent standard error of the mean.

To investigate the integrity of short-range vs. long-range connections, a frontal and a posterior cluster were created by pooling of the FP1, PP2, F7, F3, Fz, F4, F8, and P3, Pz, P4, O1, Oz, O2 electrodes, respectively [27]. Short-range and long-range connections were analyzed for the theta, upper alpha, and beta band. Preliminary analyses confirmed the absence of laterality differences for the PLI values within the clusters. Laterality was therefore disregarded as a separate factor in the analyses. This analysis could shed further light on the putative discrepancies in short- vs. long-range connectivity in neurodevelopmental disorders [20], [46]. Results of this analysis are depicted in Figure 3. Lower short-range and long-range connectivity was observed for FXS individuals compared to controls for lower alpha oscillations in the short-range frontal, *t*(18) = −3.02, *p* = .007, *η*_p_^2^ = .33, and posterior, *t*(18) = −4.20, *p* = .001, *η*_p_^2^ = .50, clusters, as well as the long-range frontal-posterior cluster, *t*(18) = −2.98, *p* = .008, *η*_p_^2^ = .33 (Figure 3, panel B). Also, posterior short-range connectivity was lower in FXS individuals than in controls for upper alpha connectivity in the posterior cluster, *t*(18) = 2.31, *p* = .033, *η*_p_^2^ = .23 (Figure 3, panel C). In contrast, functional connectivity was higher in FXS individuals than in controls for theta oscillations in the short-range posterior, *t*(18) = 2.62, *p* = .017, *η*_p_^2^ = .28, and long-range frontal-posterior cluster, *t*(18) = 2.62, *p* = .018, *η*_p_^2^ = .28 (Figure 3, panel A). This impairment of long-range functional connectivity in FXS can be particularly ascribed to networks governing alpha oscillations at rest. In addition, the current data suggest that short-range frontal and posterior connectivity for alpha oscillations is also diminished in FXS individuals. However, increased long-range frontal-posterior and short-range posterior connectivity was observed in the theta spectral band in FXS, which may be a manifestation of the alleged immature cortical network characteristics reported in FXS [27].

### Normalized graph parameters: clustering coefficient and path length

A subsequent step was to characterize the network architecture that governs neuronal oscillations using graph theory. The clustering coefficient and path length were the parameters used to characterize the local segregation (clustering) and the global integration (path length) within the network [39]. In particular, clustering is an important parameter in neurobiological systems because it highlights the functional organization of the brain, whereas the path length constitutes a valuable index of integration of networks in the brain [47].

**Figure 4:**
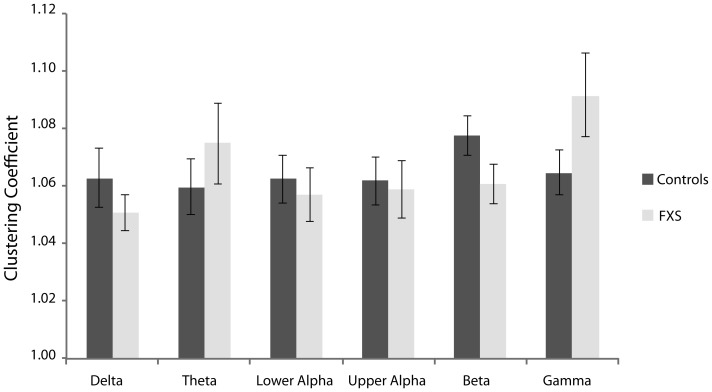
Mean normalized clustering coefficients over all epochs for FXS and controls participants in the delta (0.05–4), theta (4–8 Hz), lower alpha (8–10 Hz), upper alpha (10–13 Hz), beta (13–30 Hz), and gamma (30–45 Hz) frequency range. Error bars represent standard error of the mean.

**Figure 5:**
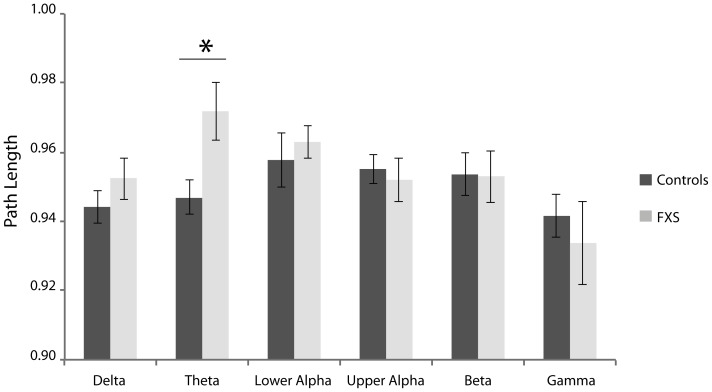
Mean normalized path length over all epochs for FXS and controls participants in the delta (0.05–4 Hz), theta (4–8 Hz), lower alpha (8–10 Hz), upper alpha (10–13 Hz), beta (13–30 Hz), and gamma (30–45 Hz) frequency range. Path length in the theta band is significant longer in FXS males as compared to controls. Asterisks represent significant differences at *p*<.05. Error bars represent standard error of the mean.

Figures 4 and 5 depict the normalized weighted clustering coefficients and normalized weighted path length, respectively, for FXS males and controls. Local segregation as represented by the clustering coefficient did not differ significantly between the groups (Figure 4). A decrease of the clustering coefficient is typically interpreted to reflect local connection loss, whereas an increase may reflect increased connectivity [48]. For normalized path length, larger normalized path length was observed for the theta spectral band in FXS individuals relative to controls, *t*(18) = 2.70, *p* = 0.02, *η*_p_^2^ = .29. The small-world index *S* for all spectral bands (except for gamma) was smaller in FXS than controls but these differences were not significant (see Table 1). This suggests that brain networks in FXS still display small-world properties. However, the differences in path length for the theta band, shows that global information transfer within the network may be particularly compromised in neuronal networks that govern theta oscillatory activity. This finding could mirror the excess of neuronal connections found in neurobiological studies [9] and could compromise the efficiency of information transfer within the network. This notion is in line with our present connectivity results and previous findings of augmented theta power activity in FXS individuals [27]. Notably, the current network parameters are not likely to be confounded by spectral power or PLI, as the normalized path length and clustering coefficients were compared to its surrogate networks indices [37].

**Table 1:** Results of the small-world index “*S*” in FXS and controls in the six EEG frequency bands.

	FXS	Controls
Delta: 0.5–4 Hz	1.10 (0.01)	1.13 (0.01)
Theta: 4–8 Hz	1.11 (0.02)	1.12 (0.01)
Alpha low: 8–10 Hz	1.10 (0.01)	1.11 (0.01)
Alpha up: 10–13 Hz	1.10 (0.01)	1.11 (0.01)
Beta: 13–30 Hz	1.11 (0.01)	1.13 (0.01)
Gamma: 30–45 Hz	1.18 (0.03)	1.13 (0.01)

Note: Values represent mean small-world index ‘*S*’. Standard error of the mean is presented between brackets.

## Discussion

Abnormalities in brain functional connectivity have been hypothesized to be a neurological hallmark of neurodevelopmental disorders [49]. Here we investigated brain functional connectivity and network topology using a resting-state EEG characterization of ongoing neuronal oscillations in fragile X syndrome males and age-matched male controls. A key finding of this study is that the previously reported augmentation in theta power and a reduction in upper alpha power [27] were matched by higher functional connectivity in the theta band, and lower functional connectivity for higher-frequency oscillations (i.e., alpha and beta). These altered neuronal oscillatory dynamics could be indicative of aberrant neuronal maturation, an interpretation that is in line with neurobiological studies showing uncontrolled synaptic overgrowth or “soft-wiring” of synaptic connections in FXS [50].

In typical brain development this synaptic growth is followed by stages of pruning and rewiring, enabling the formation of efficient structural and functional brain networks [51]. Neuronal oscillatory activity plays a critical role in the activity-dependent organization of cortical networks, and synchronization of oscillations provides an index of cortical maturation [45], [52]. This can be observed by the gradual shift from low to high frequency synchronization during development [53], [54]. In particular, delta and theta power density continues to decrease during adolescence, whereas long-range alpha synchronization shows a prolonged increase during development, processes that have been hypothesized to reflect ongoing synaptic pruning of the cortex [53]. The current connectivity findings in the alpha band show that both short-range and long-range synchrony is compromised in FXS, a putative signature of immature cortical networks.

Our functional connectivity results are furthermore adding an interesting dimension to the recently observed heightened synchrony of cortical network activity in *fmr1* knockout mice. Gonçalves and colleagues [55] observed that *fmr1* knockout mice displayed abnormally high synchrony in the firing of cortical neurons. This finding was interpreted to explain the propensity for seizures in humans with FXS. Our current findings of elevated theta driven functional connectivity suggest FXS neuronal hyperactivity may be specific for the theta band.

A second major finding was that graph theoretical analyses of the EEG resting-state data provided additional evidence of immature topological organization of neuronal networks governing theta synchronization. FXS males displayed longer path length than controls in theta driven oscillatory networks. Generally, shorter path length is believed to facilitate information transfer within the network, whereas information transfer is hampered with longer path length [22]. Although it is difficult to draw conclusions about the underlying pathophysiological aspects that cause the longer path length in FXS, it is tempting to speculate that longer path length reflects excess neuronal connectivity, resulting in uncoordinated information transfer within brain networks.

Based on the evidence of an imbalance in excitatory (glutamate) and inhibitory (GABA) neurotransmission in FXS [10], [15], we hypothesize that the current EEG spectral and network abnormalities are reflective of immature cortical networks that most likely govern theta frequency oscillations, driven by exaggerated excitatory glutamatergic circuit activity. Interestingly, there is evidence of a strong relation between the glutamatergic system and theta oscillatory activity. For example, it has been shown that glutamatergic concentration in the hippocampal region is predictive of theta activity in long-range cortical networks connecting the hippocampal region with frontal cortex [56]. Isolation of glutamatergic pathways in hippocampal areas, furthermore, eliminates theta oscillatory activity in similar neural regions [57], [58]. The current finding of higher long-range theta functional connectivity may reflect the excess functioning of the excitatory glutamatergic system in FXS. Although the underlying neurobiological mechanisms may differ from those in FXS, modeling work in Alzheimer's Disease suggested that neuronal disinhibition is a likely explanation for an initial increase in spike density of main excitatory neurons, and may furthermore account for higher incidence of epileptic activity in this disease [59]. Although the current sample of FXS participants had no history of epileptic seizure activity, evidence suggests that at least 10% of FXS males are at risk of experiencing epileptic seizures, particularly in early childhood [60]. The susceptibility to epileptic seizures has been linked to heightened neuronal circuit activity and decreased synchronous network inhibition [55], most likely caused by a failure to modulate the exaggerated glutamatergic response in the absence of FMRP. This may be a primary cause of increased neuronal circuit activity in FXS [61].

An interesting avenue for future research is to link the functional connectivity and network indices derived from the EEG resting-state to the attentional deficits frequently reported in FXS [18], [62]–[64]. For example, the current findings raise the hypothesis that the previously reported failure to habituate to sensory stimulation [32] may be a result of uncoordinated neuronal synchronization patterns [20]. Imbalanced synchronized slow and fast oscillatory activity has been found in psychiatric populations with known attentional and/or behavioral inhibitory deficits similar to those observed in FXS [19], [65], [66]. For example, increased power and synchronization in the theta band is a consistent finding in the ADHD literature [65], [66]. This EEG marker is present in both children and adults with ADHD, and is purported to reflect disturbed central nervous system functioning, causing the well-known hyperactive behavior [67]. Interestingly, it has been demonstrated that medical intervention with stimulating drugs (i.e., dexamphetamine) normalized slow wave activity in ADHD patients to a certain degree [67]. Based on the high overlap in pathophysiology and attentional deficits, FXS individuals may well benefit from therapeutic interventions that alleviate attentional deficits in ADHD patients. Indeed, a recent therapeutic intervention study using Minocycline, a tetracycline antibiotic that normalizes synaptic strength in *fMR1* knock-out mice [68], showed improved habituation to auditory stimuli in FXS individuals after they where treated with Minocycline [69]. Future studies should investigate the effects of pharmacological intervention on neuronal synchrony and network topology in the FXS brain.

Notably, the results of the current study are preliminary as they are limited by the small sample size. Although resting-state EEG recordings are relatively non-invasive, and easily acquired in typical populations, they are still difficult to obtain in individuals with a severe intellectual disability. The challenge will be to obtain larger sample sizes in future studies in order to replicate the current findings, but also to examine the implication of these resting-state EEG abnormalities for attentional, behavioral, and cognitive function in FXS. Future studies should preferably employ resting-state EEG assessments with larger electrode densities. Although a similar, or even more limited, number of electrodes have been used in previous studies, high-density EEG recordings would provide a more optimal investigation of short-range and long-range connectivity patterns, and results from graph theoretical analyses could yield a more reliable pattern of findings with regard to neural networks organization (e.g., clustering and path length).

Irrespective of these limitations, the present investigation may set the stage for an interesting line of research that could address the following questions. For example, how does the topology of the networks influence the dynamical processes that occur within the networks? How does this change during development, and how do the dynamical processes that occur within the network change the architecture of the network [22]. Based upon the current results, the topological alterations in theta driven functional connectivity may well hinder the dynamic switching between neural networks that govern sensory information processing or task-related operations, as well as neural networks that inhibit such operations. For example, it has been shown that functional brain connectivity indexed by BOLD coherence in widely distributed cortical regions is inversely related with alpha oscillatory power during the resting-state EEG [70]. Periods of high alpha power were found to disrupt BOLD coherence patterns, thus, resulting in a decrease in brain activation. This corroborates earlier findings of an inverse relation between BOLD activation with EEG oscillatory alpha power [71]–[73], and could be taken to suggest periods of neuronal inhibition. A similar pattern of findings has been found for beta oscillations, were power is inversely related to BOLD activation [74]. This could reflect the suppression of activity in sensory-motor cortices [75]. Albeit speculative, the active inhibition of cortical networks in FXS could be compromised due to diminished alpha and beta synchronization, and higher theta synchronization. The alleged aberrant dynamic switching between slow and fast oscillatory neural networks may not only be related to the activation of networks implicated in wakeful attentional processes, but may also play an important role in neural networks implicated in sleep [76]. Evidence suggests that slow-wave synchronization shows optimal small-world topologies during the sleep state [77]. Recent evidence of high neuronal synchrony in *fMR1* knockout mice was particularly evident during non-REM sleep and quiet wakefulness, and was suggested to have critical consequences for neuronal computations governing memory consolidations, and may explain the frequent sleep disturbances in FXS individuals [55], [76].

In conclusion, the current study shows that investigating the resting-state EEG in a well-defined neurodevelopmental disorder provides important information on the integrity of functional connectivity in EEG band-specific neuronal oscillations. Detailed characterization of resting-state network topologies using longitudinal investigations in future investigations, including both humans and rodents with the FXS mutation, may provide additional insights into the crucial stages in which FXS etiology compromises neural network formation. Given the evidence of distinct developmental changes in power and synchrony of neuronal oscillations in various spectral bands [45], [52], resting-state EEG investigations may aid in delineating FMR1-dependent critical periods during brain development.

## References

[1] Fu YH, Kuhl DP, Pizzuti A, Pieretti M, Sutcliffe JS, et al (1991) Variation of the CGG repeat at the fragile X site results in genetic instability: resolution of the Sherman paradox. Cell 67: 1047–1058. 1760838 10.1016/0092-8674(91)90283-5

[2] Verkerk AJ, Pieretti M, Sutcliffe JS, Fu YH, Kuhl DP, et al (1991) Identification of a gene (FMR-1) containing a CGG repeat coincident with a breakpoint cluster region exhibiting length variation in fragile X syndrome. Cell 65: 905–914. 1710175 10.1016/0092-8674(91)90397-h

[3] Oostra BA, Chiurazzi P (2001) The fragile X gene and its function. Clin Genet 60: 399–408. 11846731 10.1034/j.1399-0004.2001.600601.x

[4] Galvez R, Gopal AR, Greenough WT (2003) Somatosensory cortical barrel dendritic abnormalities in a mouse model of the fragile X mental retardation syndrome. Brain Res 971: 83–89. 12691840 10.1016/s0006-8993(03)02363-1

[5] Irwin SA, Patel B, Idupulapati M, Harris JB, Crisostomo RA, et al (2001) Abnormal dendritic spine characteristics in the temporal and visual cortices of patients with fragile-X syndrome: a quantitative examination. Am J Med Genet 98: 161–167. 11223852 10.1002/1096-8628(20010115)98:2<161::aid-ajmg1025>3.0.co;2-b

[6] McKinney BC, Grossman AW, Elisseou NM, Greenough WT (2005) Dendritic spine abnormalities in the occipital cortex of C57BL/6 Fmr1 knockout mice. Am J Med Gen part B: Neuropsych Gen 136B: 98–102. 10.1002/ajmg.b.30183 15892134

[7] Weiler IJ, Spangler CC, Klintsova AY, Grossman AW, Kim SH, et al (2004) Fragile X mental retardation protein is necessary for neurotransmitter-activated protein translation at synapses. Proc Natl Acad Sci U S A 101: 17504–17509. 15548614 10.1073/pnas.0407533101 PMC536018

[8] Pfeiffer BE, Huber KM (2007) Fragile X mental retardation protein induces synapse loss through acute postsynaptic translational regulation. J Neurosci 27: 3120–3130. 17376973 10.1523/JNEUROSCI.0054-07.2007 PMC6672463

[9] Pfeiffer BE, Huber KM (2009) The state of synapses in fragile X syndrome. Neuroscientist 15: 549–567. 19325170 10.1177/1073858409333075 PMC2762019

[10] D'Hulst C, De Geest N, Reeve SP, Van Dam D, De Deyn PP, et al (2006) Decreased expression of the GABAA receptor in fragile X syndrome. Brain Res 1121: 238–245. 17046729 10.1016/j.brainres.2006.08.115

[11] Huber K (2007) Fragile X syndrome: molecular mechanisms of cognitive dysfunction. A J Psychiatry 164: 556. 10.1176/ajp.2007.164.4.556 17403966

[12] Holcman D, Tsodyks M (2006) The emergence of Up and Down states in cortical networks. PLoS Comput Biol 2: e23. 16557293 10.1371/journal.pcbi.0020023 PMC1409813

[13] Shu Y, Hasenstaub A, McCormick DA (2003) Turning on and off recurrent balanced cortical activity. Nature 423: 288–293. 12748642 10.1038/nature01616

[14] Cooke SF, Bliss TVP (2006) Plasticity in the human central nervous system. Brain 129: 1659–1673. 16672292 10.1093/brain/awl082

[15] Bear MF, Huber KM, Warren ST (2004) The mGluR theory of fragile X mental retardation. Trends Neurosci 27: 370–377. 15219735 10.1016/j.tins.2004.04.009

[16] Hagerman RJ, Hagerman PJ (2002) Fragile X syndrome: Diagnosis, treatment, and research; Hagerman RJ, editor. Baltimore: Johns Hopkins University Press.

[17] Van der Molen MJW, Huizinga M, Huizenga HM, Ridderinkhof KR, Van der Molen MW, et al (2010) Profiling fragile X syndrome in males: strengths and weaknesses in cognitive abilities. Res Dev Disabil 31: 426–439. 19939624 10.1016/j.ridd.2009.10.013

[18] Van der Molen MJW, Van der Molen MW, Ridderinkhof KR, Hamel BCJ, Curfs LMG, et al (2012) Attentional set-shifting in fragile X syndrome. Brain Cogn 78: 206–217. 22261226 10.1016/j.bandc.2011.12.008

[19] Barry RJ, Clarke AR, Johnstone SJ (2003) A review of electrophysiology in attention-deficit/hyperactivity disorder: I. Qualitative and quantitative electroencephalography. Clin Neurophysiol 114: 171–183. 12559224 10.1016/s1388-2457(02)00362-0

[20] Uhlhaas PJ, Singer W (2012) Neuronal dynamics and neuropsychiatric disorders: toward a translational paradigm for dysfunctional large-scale networks. Neuron 75: 963–980. 22998866 10.1016/j.neuron.2012.09.004

[21] Bullmore E, Sporns O (2009) Complex brain networks: graph theoretical analysis of structural and functional systems. Nat Rev Neurosci 10: 186–198. 19190637 10.1038/nrn2575

[22] Stam CJ, van Straaten EC (2012) The organization of physiological brain networks. Clin Neurophysiol 123: 1067–1087. 22356937 10.1016/j.clinph.2012.01.011

[23] Boersma M, Smit DJ, de Bie HM, Van Baal GC, Boomsma DI, et al (2011) Network analysis of resting state EEG in the developing young brain: structure comes with maturation. Hum Brain Mapp 32: 413–425. 20589941 10.1002/hbm.21030 PMC6870229

[24] Smit DJ, Boersma M, Schnack HG, Micheloyannis S, Boomsma DI, et al (2012) The brain matures with stronger functional connectivity and decreased randomness of its network. PLoS One 7: e36896. 22615837 10.1371/journal.pone.0036896 PMC3352942

[25] Li Y, Liu Y, Li J, Qin W, Li K, et al (2009) Brain anatomical network and intelligence. PLoS Comput Biol 5: e1000395. 19492086 10.1371/journal.pcbi.1000395 PMC2683575

[26] Van den Heuvel MP, Stam CJ, Kahn RS, Hulshoff Pol HE (2009) Efficiency of functional brain networks and intellectual performance. J Neurosci 29: 7619–7624. 19515930 10.1523/JNEUROSCI.1443-09.2009 PMC6665421

[27] Van der Molen MJW, Van der Molen MW (2013) Reduced alpha and exaggerated theta power during the resting-state EEG in fragile X syndrome. Biol Psychol 92: 216–219. 23182872 10.1016/j.biopsycho.2012.11.013

[28] Raven J, Court JH (1998) Standard Progressive Matrices, Raven Manual: Section 3. Oxford: Oxford Psychologists Press.

[29] Snijders JT, Tellegen PJ, Laros JA (1998) Snijders-Oomen Niet-verbale intelligentietest. Verantwoording en handleiding [Snijders-Oomen Non-verbal intelligence test. Justification and manual]: Groningen: Wolters-Noordhoff.

[30] Schlichting L (2004) Peabody picture vocabulary test III-NL. Nederlandse versie [Dutch Version]: Amsterdam: Harcourt Assessment.

[31] Van der Molen MJW, Van der Molen MW, Ridderinkhof KR, Hamel BC, Curfs LM, et al (2012) Auditory and visual cortical activity during selective attention in fragile X syndrome: a cascade of processing deficiencies. Clin Neurophysiol 123: 720–729. 21958658 10.1016/j.clinph.2011.08.023

[32] Van der Molen MJW, Van der Molen MW, Ridderinkhof KR, Hamel BC, Curfs LM, et al (2012) Auditory change detection in fragile X syndrome males: A brain potential study. Clin Neurophysiol 123: 1309–1318. 22192499 10.1016/j.clinph.2011.11.039

[33] Stam CJ, Nolte G, Daffertshofer A (2007) Phase lag index: assessment of functional connectivity from multi channel EEG and MEG with diminished bias from common sources. Hum Brain Mapp 28: 1178–1193. 17266107 10.1002/hbm.20346 PMC6871367

[34] Montez T, Linkenkaer-Hansen K, van Dijk BW, Stam CJ (2006) Synchronization likelihood with explicit time-frequency priors. Neuroimage 33: 1117–1125. 17023181 10.1016/j.neuroimage.2006.06.066

[35] Peraza LR, Asghar AU, Green G, Halliday DM (2012) Volume conduction effects in brain network inference from electroencephalographic recordings using phase lag index. J Neurosci Methods 207: 189–199. 22546477 10.1016/j.jneumeth.2012.04.007

[36] Stam CJ, van Straaten EC (2012) Go with the flow: use of a directed phase lag index (dPLI) to characterize patterns of phase relations in a large-scale model of brain dynamics. Neuroimage 62: 1415–1428. 22634858 10.1016/j.neuroimage.2012.05.050

[37] Stam CJ, de Haan W, Daffertshofer A, Jones BF, Manshanden I, et al (2009) Graph theoretical analysis of magnetoencephalographic functional connectivity in Alzheimer's disease. Brain 132: 213–224. 18952674 10.1093/brain/awn262

[38] Stam CJ, Jones BF, Nolte G, Breakspear M, Scheltens P (2007) Small-world networks and functional connectivity in Alzheimer's disease. Cereb Cortex 17: 92–99. 16452642 10.1093/cercor/bhj127

[39] Watts DJ, Strogatz SH (1998) Collective dynamics of ‘small-world’ networks. Nature 393: 440–442. 9623998 10.1038/30918

[40] Latora V, Marchiori M (2001) Efficient behavior of small-world networks. Phys Rev Letters 87: 198701. 10.1103/PhysRevLett.87.198701 11690461

[41] Dijkstra EW (1959) A note on two problems in connexion with graphs. Num Mathematik 1: 269–271.

[42] Van Steen M (2010) Graph Theory and Complex Networks: An Introduction. Amsterdam

[43] Sporns O (2006) Small-world connectivity, motif composition, and complexity of fractal neuronal connections. Biosystems 85: 55–64. 16757100 10.1016/j.biosystems.2006.02.008

[44] Humphries MD, Gurney K (2008) Network ‘small-world-ness’: a quantitative method for determining canonical network equivalence. PLoS One 3: e0002051. 18446219 10.1371/journal.pone.0002051 PMC2323569

[45] Uhlhaas PJ, Roux F, Singer W, Haenschel C, Sireteanu R, et al (2009) The development of neural synchrony reflects late maturation and restructuring of functional networks in humans. Proc Natl Acad Sci U S A 106: 9866–9871. 19478071 10.1073/pnas.0900390106 PMC2687997

[46] Murias M, Webb SJ, Greenson J, Dawson G (2007) Resting state cortical connectivity reflected in EEG coherence in individuals with autism. Biol Psychiatry 62: 270–273. 17336944 10.1016/j.biopsych.2006.11.012 PMC2001237

[47] Sporns O (2011) Networks of the Brain; Sporns O, editor. Cambridge, Massachusetts: The MIT Press.

[48] Supekar K, Menon V, Rubin D, Musen M, Greicius MD (2008) Network analysis of intrinsic functional brain connectivity in Alzheimer's disease. PLoS Comput Biol 4: e1000100. 18584043 10.1371/journal.pcbi.1000100 PMC2435273

[49] Dierssen M, Ramakers GJ (2006) Dendritic pathology in mental retardation: from molecular genetics to neurobiology. Genes Brain Behav 5 Suppl 2: 48–60. 16681800 10.1111/j.1601-183X.2006.00224.x

[50] Tessier CR, Broadie K (2009) Activity-dependent modulation of neural circuit synaptic connectivity. Front Mol Neurosci 2: 8. 19668708 10.3389/neuro.02.008.2009 PMC2724028

[51] Supekar K, Musen M, Menon V (2009) Development of large-scale functional brain networks in children. PLoS Biol 7: e1000157. 19621066 10.1371/journal.pbio.1000157 PMC2705656

[52] Uhlhaas PJ, Roux F, Rodriguez E, Rotarska-Jagiela A, Singer W (2010) Neural synchrony and the development of cortical networks. Trends Cogn Sci 14: 72–80. 20080054 10.1016/j.tics.2009.12.002

[53] Campbell IG, Feinberg I (2009) Longitudinal trajectories of non-rapid eye movement delta and theta EEG as indicators of adolescent brain maturation. Proc Natl Acad Sci U S A 106: 5177–5180. 19307577 10.1073/pnas.0812947106 PMC2664015

[54] Whitford TJ, Rennie CJ, Grieve SM, Clark CR, Gordon E, et al (2007) Brain maturation in adolescence: concurrent changes in neuroanatomy and neurophysiology. Hum Brain Mapp 28: 228–237. 16767769 10.1002/hbm.20273 PMC6871488

[55] Gonçalves JT, Anstey JE, Golshani P, Portera-Cailliau C (2013) Circuit level defects in the developing neocortex of Fragile X mice. Nat Neurosci 16: 903–911. 23727819 10.1038/nn.3415 PMC3695061

[56] Gallinat J, Kunz D, Senkowski D, Kienast T, Seifert F, et al (2006) Hippocampal glutamate concentration predicts cerebral theta oscillations during cognitive processing. Psychopharmacology (Berl) 187: 103–111. 16767420 10.1007/s00213-006-0397-0

[57] Basar E, Guntekin B (2008) A review of brain oscillations in cognitive disorders and the role of neurotransmitters. Brain Res 1235: 172–193. 18640103 10.1016/j.brainres.2008.06.103

[58] Buzsaki G, Draguhn A (2004) Neuronal oscillations in cortical networks. Science 304: 1926–1929. 15218136 10.1126/science.1099745

[59] de Haan W, Mott K, van Straaten EC, Scheltens P, Stam CJ (2012) Activity dependent degeneration explains hub vulnerability in Alzheimer's disease. PLoS Comput Biol 8: e1002582. 22915996 10.1371/journal.pcbi.1002582 PMC3420961

[60] Berry-Kravis E (2002) Epilepsy in fragile X syndrome. Dev Med Child Neurol 44: 724–728. 12418611 10.1017/s0012162201002833

[61] Hagerman PJ, Stafstrom CE (2009) Origins of epilepsy in fragile X syndrome. Epilepsy Curr 9: 108–112. 19693328 10.1111/j.1535-7511.2009.01309.x PMC2728488

[62] Cornish K, Cole V, Longhi E, Karmiloff-Smith A, Scerif G (2012) Does attention constrain developmental trajectories in fragile x syndrome? A 3-year prospective longitudinal study. Am J Int Dev Dis 117: 103–120. 10.1352/1944-7558-117.2.103 22515826

[63] Scerif G, Cornish K, Wilding J, Driver J, Karmiloff-Smith A (2007) Delineation of early attentional control difficulties in fragile X syndrome: focus on neurocomputational changes. Neuropsychologia 45: 1889–1898. 17254617 10.1016/j.neuropsychologia.2006.12.005 PMC2613507

[64] Scerif G, Longhi E, Cole V, Karmiloff-Smith A, Cornish K (2012) Attention across modalities as a longitudinal predictor of early outcomes: the case of fragile X syndrome. J Child Psych Psychiatry 53: 641–650. 10.1111/j.1469-7610.2011.02515.x 22211574

[65] Barry RJ, Clarke AR, Johnstone SJ, McCarthy R, Selikowitz M (2009) Electroencephalogram theta/beta ratio and arousal in attention-deficit/hyperactivity disorder: evidence of independent processes. Biol Psychiatry 66: 398–401. 19500774 10.1016/j.biopsych.2009.04.027

[66] Bresnahan SM, Barry RJ (2002) Specificity of quantitative EEG analysis in adults with attention deficit hyperactivity disorder. Psychiatry Res 112: 133–144. 12429359 10.1016/s0165-1781(02)00190-7

[67] Bresnahan SM, Barry RJ, Clarke AR, Johnstone SJ (2006) Quantitative EEG analysis in dexamphetamine-responsive adults with attention-deficit/hyperactivity disorder. Psychiatry Res 141: 151–159. 16343642 10.1016/j.psychres.2005.09.002

[68] Bilousova TV, Dansie L, Ngo M, Aye J, Charles JR, et al (2009) Minocycline promotes dendritic spine maturation and improves behavioural performance in the fragile X mouse model. J Med Genet 46: 94–102. 18835858 10.1136/jmg.2008.061796

[69] Schneider A, Jacena ML, Patrick A, Rawi N, Tasleem C, et al (In press) Electrocortical changes associated with minocycline treatment in fragile X syndrome. J Psychopharmacol 10.1177/0269881113494105 PMC4962861 23981511

[70] Tagliazucchi E, von Wegner F, Morzelewski A, Brodbeck V, Laufs H (2012) Dynamic BOLD functional connectivity in humans and its electrophysiological correlates. Front Hum Neurosci 6: 339. 23293596 10.3389/fnhum.2012.00339 PMC3531919

[71] Goncalves SI, de Munck JC, Pouwels PJ, Schoonhoven R, Kuijer JP, et al (2006) Correlating the alpha rhythm to BOLD using simultaneous EEG/fMRI: inter-subject variability. Neuroimage 30: 203–213. 16290018 10.1016/j.neuroimage.2005.09.062

[72] Laufs H, Kleinschmidt A, Beyerle A, Eger E, Salek-Haddadi A, et al (2003) EEG-correlated fMRI of human alpha activity. Neuroimage 19: 1463–1476. 12948703 10.1016/s1053-8119(03)00286-6

[73] Laufs H, Krakow K, Sterzer P, Eger E, Beyerle A, et al (2003) Electroencephalographic signatures of attentional and cognitive default modes in spontaneous brain activity fluctuations at rest. Proc Natl Acad Sci U S A 100: 11053–11058. 12958209 10.1073/pnas.1831638100 PMC196925

[74] Ritter P, Moosmann M, Villringer A (2009) Rolandic alpha and beta EEG rhythms' strengths are inversely related to fMRI-BOLD signal in primary somatosensory and motor cortex. Hum Brain Mapp 30: 1168–1187. 18465747 10.1002/hbm.20585 PMC6870597

[75] Pfurtscheller G, Lopes da Silva FH (1999) Event-related EEG/MEG synchronization and desynchronization: basic principles. Clin Neurophysiol 110: 1842–1857. 10576479 10.1016/s1388-2457(99)00141-8

[76] Miano S, Bruni O, Elia M, Scifo L, Smerieri A, et al (2008) Sleep phenotypes of intellectual disability: a polysomnographic evaluation in subjects with Down syndrome and Fragile-X syndrome. Clin Neurophysiol 119: 1242–1247. 18417419 10.1016/j.clinph.2008.03.004

[77] Ferri R, Rundo F, Bruni O, Terzano MG, Stam CJ (2007) Small-world network organization of functional connectivity of EEG slow-wave activity during sleep. Clin Neurophysiol 118: 449–456. 17174148 10.1016/j.clinph.2006.10.021

